# The Human Footprint in Mexico: Physical Geography and Historical Legacies

**DOI:** 10.1371/journal.pone.0121203

**Published:** 2015-03-24

**Authors:** Charlotte González-Abraham, Exequiel Ezcurra, Pedro P. Garcillán, Alfredo Ortega-Rubio, Melanie Kolb, Juan E. Bezaury Creel

**Affiliations:** 1 Centro de Investigaciones Biológicas del Noroeste, Programa de Planeación Ambiental y Conservación, La Paz, Baja California Sur, Mexico; 2 University of California Institute for Mexico and United States, Riverside, California, United States of America; 3 Comisión Nacional para el Conocimiento y Uso de la Biodiversidad, Mexico, DF, Mexico; 4 The Nature Conservancy, Mexico, DF, Mexico; University of California Davis, UNITED STATES

## Abstract

Using publicly available data on land use and transportation corridors we calculated the human footprint index for the whole of Mexico to identify large-scale spatial patterns in the anthropogenic transformation of the land surface. We developed a map of the human footprint for the whole country and identified the ecological regions that have most transformed by human action. Additionally, we analyzed the extent to which (a) physical geography, expressed spatially in the form of biomes and ecoregions, compared to (b) historical geography, expressed as the spatial distribution of past human settlements, have driven the patterns of human modification of the land. Overall Mexico still has 56% of its land surface with low impact from human activities, but these areas are not evenly distributed. The lowest values are on the arid north and northwest, and the tropical southeast, while the highest values run along the coast of the Gulf of Mexico and from there inland along an east-to-west corridor that follows the Mexican transversal volcanic ranges and the associated upland plateau. The distribution of low- and high footprint areas within ecoregions forms a complex mosaic: the generally well-conserved Mexican deserts have some highly transformed agro-industrial areas, while many well-conserved, low footprint areas still persist in the highly-transformed ecoregions of central Mexico. We conclude that the spatial spread of the human footprint in Mexico is both the result of the limitations imposed by physical geography to human development at the biome level, and, within different biomes, of a complex history of past civilizations and technologies, including the 20^th^ Century demographic explosion but also the spatial pattern of ancient settlements that were occupied by the Spanish Colony.

## Introduction

Ecological patterns and processes are influenced by human activities in two main ways: directly, by the transformation of land into infrastructure and productive areas [1], or indirectly, through the byproducts of human activities that might disperse away from their causal source and degrade ecosystem functions [2]. Direct modifications of the land through human infrastructure (human settlements, transportation pathways, and power lines) and productive areas (agriculture, aquaculture, forestry, and cattle ranching) have increased globally during the last century as a result of accelerated human population growth [1,3,4].

Several studies have analyzed the patterns of direct human modification of the land surface as a proxy of human influence on natural ecosystems (e.g., [5–10]). Although human modification indices do not convey the entire human effect expressed as changes historically accumulated over natural ecosystems, they are useful to infer the spatial pattern and extent of the capacity of humans to transform the earth through land use [11]. Many studies analyze how diverse ecological regions have different capacity to respond to landscape transformations (e.g., [12,13]), but only a few of them (e.g., [14–16]) analyze how the physical geography (defined, for example, as biomes or ecoregions) affects the spatial patterns of human modification.

Mexico is an ecologically heterogeneous country that hosts a diverse array of ecosystems ranging from hyper-arid deserts to tropical rainforests, which have evolved as a consequence of both the country’s complex topography and its particular location between the Nearctic and Neotropical biogeographic realms. Mexico is also one of the biologically megadiverse countries of the world, with high endemism for birds, mammals, and reptiles [17].

In principle, it would be expected that human developments and land transformations in Mexico follow the country’s complex environmental mosaic, with regions where environmental conditions are more favorable for human settlement and occupation (given a particular level of technological development) showing a larger human footprint. But land settlements and landscape transformations are not only the result of physical geography; there are also technological and historical components, linked to the ability of different societies to use different environments, which can be especially important to understand land-use change in regions that have harbored dense settlements for millennia [18,19,20]. This historic dimension is especially relevant in countries such as Mexico, with a long history of human occupation and well-documented civilization collapses. Indeed, despite the common misperception that Europeans found a New World that was largely unoccupied and wild, what Spaniards found in Mexico was a densely populated territory with well-developed agricultural settlements and large urban centers that heavily impacted their respective hinterlands. When Europeans arrived to Mesoamerica the population of the larger territory of what we now call Mexico was in the order of tens of millions of people [21,22,23]. Although the native population was devastated by European diseases, the *encomienda* system, and 16^th^ Century droughts [24,25], its geographical distribution at the time of Spanish conquest conditioned the subsequent land occupation and landscape transformations. Even livestock husbandry developed initially in dryland agricultural areas that had been abandoned by the population collapse [26].

The ability of humans to transform the face of the earth has been referred to as the ¨human footprint.¨ In 2002 Sanderson et al. developed a geographically-explicit index that displays in map form the sum of all visible anthropogenic transformations on a large territory [6]. Sanderson et al.’s Human Footprint Index (*HF*) is calculated by adding all major large-scale anthropogenic transformations over the land surface. It uses four variables to summarize the effects of human modification: population density, land use change, access areas, and electric infrastructure. This index has been used and modified in different studies and at different scales (e.g., [9,27]), but always following the main idea that the intensity of human influence is the result of the type of activity, the area that each activity occupies, and the accumulation of activities within large areas [6,9]. Its values distributed on a map reveal the major patterns of human influence over the broad landscape [6–9,27]. The advantages of the *HF* index lie in the fact that it uses publicly available geographic data for the majority of countries and hence it is easily reproducible by different researchers in different regions [8,27,28], and its calculations are statistically simple, with an explicative clarity that can be easily understood [6].

In this study we present a map of the Human Footprint for Mexico, basically following the methodology of Sanderson et al. [6]. Our goals were manifold. Because Mexico is a country with a rich historical legacy and a prolific literature on its profound landscape transformations, we wanted first to analyze how much of a complex set of process and patterns that have been described mostly qualitatively coincided with the quantitative results of the Human Footprint approach. Secondly, we wanted to analyze how much of the variation in Human Footprint across the country is related to environmental variation at different scales, including (a) the effect of large-scale climate in biomes such as deserts or tropical rainforests, (b) the effect of different ecological regions within the large biomes, and (c) variation within ecoregions. Thirdly, we aimed at testing the power of the approach to identify the size and number of extant patches of well-preserved ecosystems that may inform future initiatives of biodiversity conservation. Finally, we analyzed how Human Footprint values correlate spatially with regions of historically-intense land use.

## Methods

We selected spatial datasets that represent, as much as possible, all the different sources of direct human modification of the land surface: human settlements, cultivated land (agriculture, forestry plantations and cultivated grasslands), cultivated coasts (marine aquaculture) and roads.

### Selection of databases and spatial resolution

We used digital vector maps from Mexico’s National Institute of Statistics and Geography (*Instituto Nacional de Geografía*, *Estadística*, *e Informática*—INEGI), and complemented them with road maps from Mexico’s Institute of Transport (*Instituto Mexicano del Transporte*—IMT). INEGI’s [29] maps of land use and land cover (series III, at a scale of 1:250,000), were used to obtain spatial distribution of urban settlements, agriculture, aquaculture, forestry plantations, and cultivated grassland areas. The population numbers of non-urban settlements (<2500 persons) were included based on the 2010 INEGI’s population census. Major roads, highways, and local dirt roads were obtained from two sources: (a) the Mexican Institute of Transport digital road map [30], and (b) the ESRI Mexican roads database [31], both at a scale 1:200,000. In order to reduce the risk of small-scale mapping errors we chose an analytical resolution of 500 m (a cell of 500 m × 500 m).

To map urban areas and population clusters we used vector data from INEGI’s [29] land use and land cover map selecting all those areas identified as corresponding to urban settlements (>2500 people). Data for smaller, non-urban settlements (<2500) were generated from the 2010 INEGI Census, which displays information as “locations” (data-points inhabited by one or more households) with the number of people living in it.

### Assignment of scores

Human modification at any given location is defined by two factors: intensity and extent [9]. Intensity is the degree to which an activity at a location has transformed the original ecosystem. We incorporated the intensity factor through the assignment of scores to the sources of direct human modification. Extent measures the aerial extension of the human activity at a specific location. We incorporated the extent through a threshold decision: due to our resolution level (500 m) and the difficulty to rigorously estimate the areal extent of each human transformation inside each cell, we used a presence/absence criterion in each pixel, considering an activity as present in a cell if it occupied at least half of the cell.

To map an index of population density we selected all urban areas as high population-density areas and gave them a maximum score of 10 (Table 1). Smaller locations, which appear as a point in the map, were ascribed to a single grid cell. Cells with more than 2500 inhabitants were lumped within the urban category; cells with 500–2500 inhabitants received a score of 7, while cells with less than 500 inhabitants received a score of 5.

**Table 1:** Scores of human transformation.

Proxies	HT Score
Urban	10
Rural (500–2499 inhabitants)	7
Rural (< 500 inhabitants)	5
Agriculture	7
Marine aquaculture	7
Forestry plantations	5
Cultivated and natural grasslands	5
Paved roads	7
Dirt roads	5

The scores were assigned considering the irreversibility of the human transformation on the land surface and based on other published human footprint studies (e.g., [6,7,9,30,35]).

Other land use types (agriculture, cultivated grasslands, forestry plantations, and aquaculture) were extracted from INEGI’s land use and land cover map and each was saved as a separate layer. Following Sanderson et al. [6], we assigned a score ranging from 0 (low) to 10 (high) to each category of human transformation of the land surface (agriculture, cultivated or induced grasslands, roads, aquaculture, forestry plantations, and urban areas) based on the irreversibility of the transformation as used in other similar studies (e.g., [6,7,9,27,32]). Agriculture and aquaculture, for example, which destroy the native land cover but maintain some functionally in the substrate were given a score of 7, while cultivated grasslands and forestry plantations, which maintain a more permanent ground cover and some of the native flora, received a score of 5, and urban areas, which irreversibly destroy the native land cover and most of the soil substrate, received a score of 10.

Roads and transportation corridors were divided into two general categories that differ on their relative land modification: paved and dirt roads. Roads in the databases are represented in the original maps as elements of linear dimension, i.e., their area equals zero. We transformed them to areal dimensions generating a 250 m buffer on each side of paved roads, and 100 m on each side of dirt roads. Based on the different degree of land transformation of paved and dirt roads [33,34], we assigned a score of 5 to dirt roads that covered more than half of each grid cell, and 8 to paved roads (we did not include navigable rivers or railway lines in our analysis because they do not play a significant role as transportation corridors in Mexico). The number of lanes and traffic density of roads were not included in the calculation of our scores, as that information is not available in the databases we consulted.

### Overall estimate of direct human modification

To calculate the overall effect of land use and infrastructure, we converted all vector maps with the different human sources of land transformation to a raster format with a pixel size of 500 m × 500 m, with their respective human modification scores for each pixel. According to our threshold criterion, only one activity can be present in each cell, except for roads, to which we allow their overlapping with the rest of activities. Therefore, roads might coexist within a single pixel with other forms of land transformation, and adding all layers could potentially exceed the maximum score of 10. In practical terms, this means that roads necessarily have a different additive effect on the footprint within a given cell: In a pristine, little transformed ecosystem the impact of a new dirt road will be much higher than the impact of a similar road in, say, a grazed grassland.

In order to address this problem we used Theobald’s [9] fuzzy algebraic sum of human transformation scores to reduce errors due to partial dependence between layers that can coincide in the same pixel. This method first rescales the scores on a scale from 0 to 1 assuming that pixels with more than one layer should have higher human modification than those pixels with only one layer. Thus, the overall value of direct human transformation *HT*_*i*_ (on a scale from 0 to 1) at each pixel *i* is calculated as $HT_{i}=1-\prod_{j=1}^{9}\left(1-h_{ij}\right)$, where *h*_*ij*_ represents the human modification score of each individual source *j* present in pixel *i*. The values of *HT*_*i*_ range between 0 and a maximum of 1, imposing a realistic upper limit to the added *HT* values. Finally, we re-scaled to range between 0 and 10 for our presentation.

### The human footprint map

Finally, we smoothed the map for pixel neighborhood effects, averaging the value of each cell with those of its direct neighbors (i.e., a 3 × 3 cell neighborhood moving average algorithm). We defined the resulting map as the human footprint, an estimate of the spatial patterns of the direct human modification on Mexico’s land surface with quantitative scores *HF*_*i*_ ranging from 0 to 10 for each pixel *i*, where zero represents an extremely low level of direct modification of land and 10 represents areas where maximum accumulation of human transformation have occurred. It is important to have in mind that under this moving average algorithm the value of 0 in a cell requires that all neighboring cells also had a value of 0.

### Physical geography and the human footprint

We used the map of ecological regions of North America [35], officially used by INEGI in Mexico. Level I in this map defines large-scale regions based on broad continental-wide patterns of physical geography, climate, and vegetation physiognomy, similar in scale and scope to the ecological concept of biomes. Level II is hierarchically nested within level I and provides ecoregional subdivisions within the larger biomes in terms of distinct sub-continental traits in local physiography, biogeographic history, and biodiversity and endemism [35]. For our study, we modified the level II map in order to achieve the same cartographic detail in the Mexican drylands as in the rest of the country by separating the Warm Deserts category into three distinct ecoregions: Sonoran Desert, Chihuahuan Desert, and Potosí High Plateau (*Altiplano Potosino*). We explored how the physical geography (expressed in the ecoregions and biomes maps) influences the geographical pattern of the human footprint. To characterize the geographical pattern of *HF* we calculated: (a) the statistical distribution properties (mean, standard deviation, and skewness) of the human footprint within each ecoregion and each biome, and (b) the mean patch size and percentage of areas within each ecoregion with minimal direct human influence (*HF* = 0), as an indicator of the size of large continuous areas with extant native vegetation cover. Finally, we decomposed the overall variance in the human footprint into three ANOVA components: (a) variance explained by differences in *HF* between biomes, (b) variance explained by differences in *HF* between ecoregions nested-within-biomes, and (c) residual variance, or variance within ecoregions. Because the pixel-level information had been averaged with that of its eight neighbors (except in coastal pixels where the number of neighbors is lower), we conservatively took the total number of 3 × 3 grid cell units as our degrees of freedom for the ANOVA analysis (i.e., that is, although the original rasterized map has 7 793 854 grid cells, we took our degrees of freedom as 865 984).

### Pre-Hispanic settlements and the human footprint

Finally, we tested whether there was a spatial association between the presence of ancient pre-Hispanic settlements and the modern human footprint. In order to do this, we first downloaded the coordinates of Mexico’s 183 main archaeological sites, compiled by the National Institute for Anthropology and History (INAH; http://www.geoportal.inah.gob.mx/). This list was complemented by the lists of major large pre-Hispanic settlements as identified by Kellog [36] for central Mexico, Sharer [37] for the Maya region, and Whitmore and Turner [38] for both. We overlaid this pre-Hispanic map on the ecoregional map of Mexico, and identified the ecoregions that harbored in the past many and large settlements. Then, using a *t*-test for comparison among means, we tested the hypothesis that ecoregions that had sheltered intense pre-Hispanic occupation show currently a higher mean *HF* value than those regions that did not harbor dense occupation (because the process involves multiple comparisons, the *t*-test probabilities were adjusted using a Bonferroni correction).

## Results

### General description of the human footprint in Mexico

**Fig 1:**
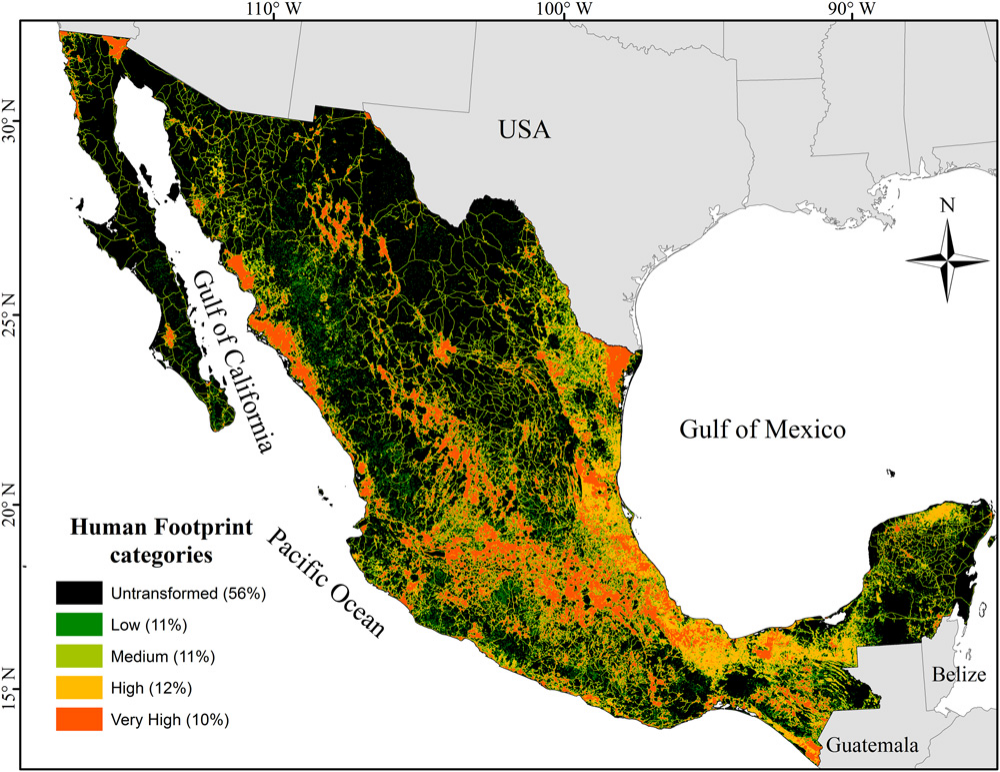
Human footprint map. The areas in black show with the highest human footprint values in a well-defined spatial pattern. They run along the coastal plains of the Gulf of Mexico and from there inland along an east-to-west corridor that follows the Mexican transversal volcanic ranges and the associated upland plateau. The areas in black represent the lowest values of human footprint located on Mexico’s arid northwest and its tropical southeast (chiefly the Yucatán peninsula).

Half of the country (55.9% of the total land surface) fell within the very low human footprint value (*HF* = 0), implying that over half of Mexico still maintains vegetation cover in reasonably good environmental conditions. Only 10.3% was classified within the very high footprint value (*HF* ≥ 7; Fig. 1). The remaining 33.8% belongs to intermediate values: low footprint (*HF* = 0.5–1) with 11.2% of the territory; medium (*HF* = 2–3) with 10.6%, and high (*HF* = 4–6) with 12%.

The areas with the highest human footprint values show a well-defined spatial pattern (Fig. 1): They run along the coastal plains of the Gulf of Mexico and from there inland along an east-to-west corridor that follows the Mexican transversal volcanic ranges and the associated upland plateau. Areas of high footprint are also visible along the eastern coast of the Gulf of California. The rest of Mexico’s arid northwest and its tropical southeast (chiefly the Yucatán peninsula) present relatively low *HF* values. The country’s south (i.e., the region south of the transversal volcanic axis to the Pacific coast) harbors a fragmented mosaic of low and high footprint values.

### Biomes, ecoregions, and the human footprint

**Fig 2:**
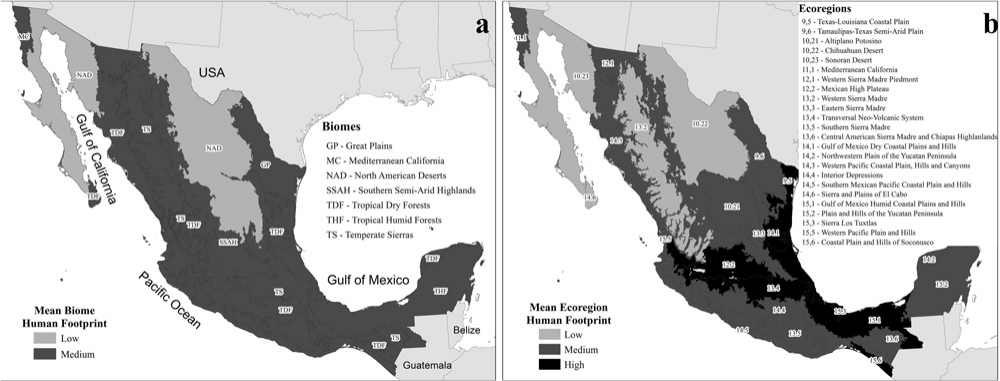
Mean footprint in large regions. Biomes (a) and ecoregions (b) of Mexico grouped into categories defined by their mean human footprint (low, medium, and high human footprint, with mean *HF*<1; 1<*HF*<3, and *HF*>3, respectively).

**Fig 3:**
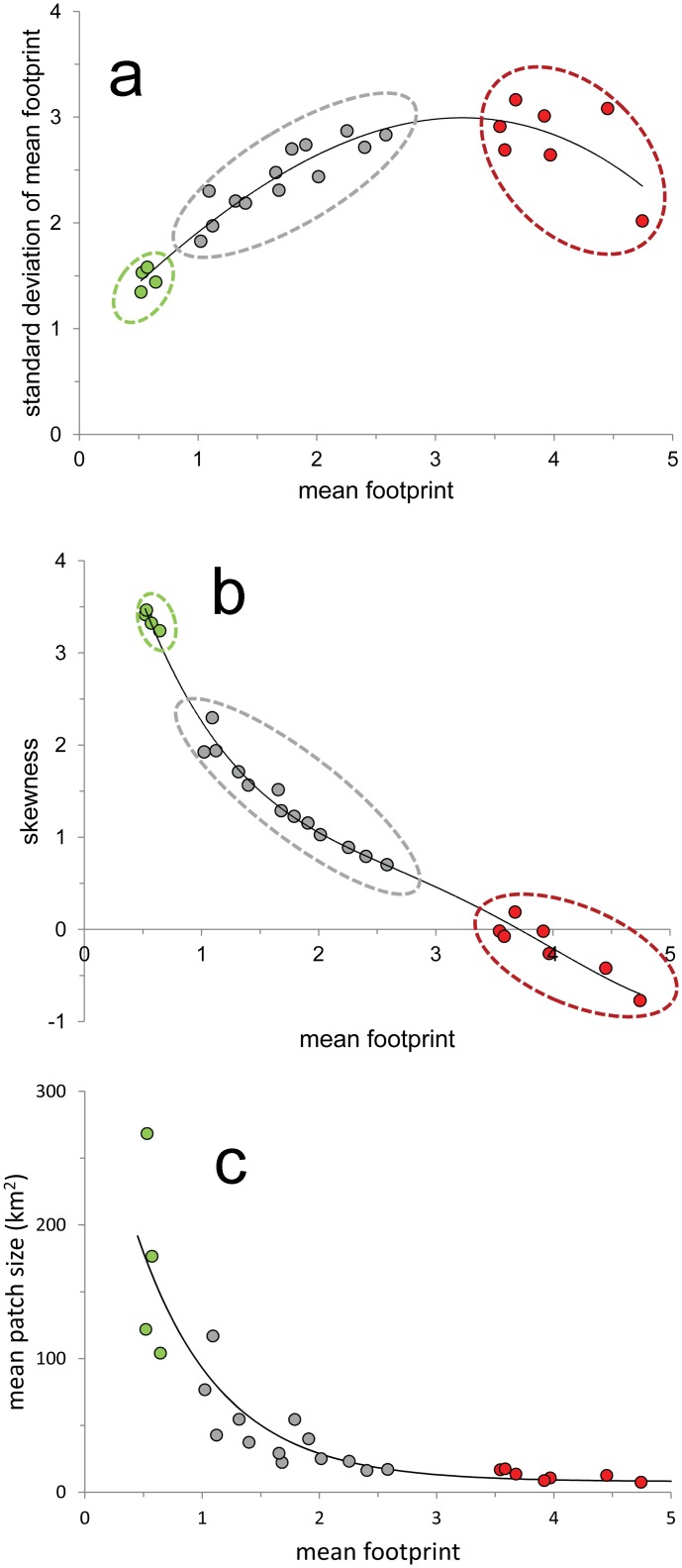
Ecoregional footprint nested within biomes. Large differences in ecoregional *HF* values (white points) were observed within most biomes (black points). Biomes names as follow Great Plains (GP), Mediterranean California (MC), North American Deserts (NAD), Southern Semi-Arid Highlands (SSAH), Temperate Sierras (TS), Tropical Dry Forests (TDF) and Tropical Humid Forests (THF).

The distribution of mean *HF* values in Mexico’s biomes and ecoregions is given in Figs. 2 and 3, and Table 2. The coastal Sierra Los Tuxtlas (an ecoregion within the Tropical Humid Forest biome where most of the original tropical forest has been cut to open way for pastures) showed the highest mean *HF* (4.74), while the Western Sierra Madre (within the Temperate Sierras biome) had the lowest mean *HF* value (0.52; see Table 2). Only two ecoregions, the Chihuahuan and Sonoran deserts, showed more than 80% of their total area falling within the low footprint category. On the other extreme, there were seven ecoregions that had a large proportion of their area in the high footprint categories (Table 2).

**Table 2:** Ecoregions of Mexico ordered according to their mean footprint value.

Ecoregions	Biomes	Mean *HF*	st. dev.	skew.	mean patch size (km^2^)	low *HF* (%)
**Low-footprint ecoregions**						
Western Sierra Madre	Temperate Sierras	**0.52**	1.35	3.42	121	75
Chihuahuan Desert	North American Deserts	**0.53**	1.53	3.46	268	82
Sonoran Desert	North American Deserts	**0.57**	1.58	3.32	176	81
Sierra and Plains of El Cabo	Tropical Dry Forests	**0.64**	1.44	3.24	104	68
**Medium-footprint ecoregions**						
Plain and Hills of the Yucatan Peninsula	Tropical Humid Forests	1.02	1.83	1.93	76	64
Mediterranean California	Mediterranean California	1.09	2.30	2.30	116	71
Southern Sierra Madre	Temperate Sierras	1.12	1.97	1.94	42	58
Altiplano Potosino	North American Deserts	1.32	2.21	1.71	54	60
Eastern Sierra Madre	Temperate Sierras	1.40	2.19	1.57	37	54
Northwestern Plain of the Yucatan Peninsula	Tropical Dry Forests	1.66	2.48	1.57	29	54
Central American Sierra Madre and Chiapas Highlands	Temperate Sierras	1.68	2.31	1.29	22	44
Western Sierra Madre Piedmont	Southern Semi-Arid Highlands	1.79	2.70	1.23	54	58
Western Pacific Coastal Plain, Hills and Canyons	Tropical Dry Forests	1.91	2.74	1.15	40	52
Tamaulipas-Texas Semi-Arid Plain	Great Plains	2.01	2.44	1.03	25	42
Western Pacific Plain and Hills	Tropical Humid Forests	2.25	2.87	0.89	23	46
Southern Mexican Pacific Coastal Plain and Hills	Tropical Dry Forests	2.40	2.71	0.79	16	37
Interior Depressions	Tropical Dry Forests	2.58	2.83	0.70	17	35
**High-footprint ecoregions**						
Coastal Plain and Hills of Soconusco	Tropical Humid Forests	3.54	2.91	-0.2	16	27
Gulf of Mexico Humid Coastal Plains and Hills	Tropical Humid Forests	3.58	2.69	-0.07	17	23
Transversal Neo-Volcanic System	Temperate Sierras	3.68	3.16	0.19	13	25
Mexican High Plateau	Southern Semi-Arid Highlands	3.92	3.01	-0.02	8	19
Gulf of Mexico Dry Coastal Plains and Hills	Tropical Dry Forests	3.97	2.64	-0.26	10	23
Texas-Louisiana Coastal Plain	Great Plains	4.45	3.08	-0.42	12	22
Sierra Los Tuxtlas	Tropical Humid Forests	4.74	2.02	-0.77	7	5

The standard deviation and the skewness of the *HF* distribution within each ecoregion are also given, together with the mean patch size (in km^2^) of very low-footprint areas (*HF* = 0) and the percentage of area with very low *HF*.

The mean *HF* value at the ecoregional level varied between 0.52 and 4.74, and large differences in ecoregional *HF* values were observed within most biomes (Table 2, and Fig. 3). Only the North American Deserts biome had most of its ecoregions with low mean *HF* values (0.53 for the Chihuahuan Desert, and 0.57 for the Sonoran Desert). The Mediterranean California biome has no ecoregional variation because it contains only one ecoregion in Mexico. The rest of the biomes showed marked differences in mean *HF* values between their ecoregions (Fig. 3). Except for the North American Desert biome and Mediterranean California, the rest of the biomes contained at least one ecoregion with *HF* > 3 (Table 2).

**Fig 4:**
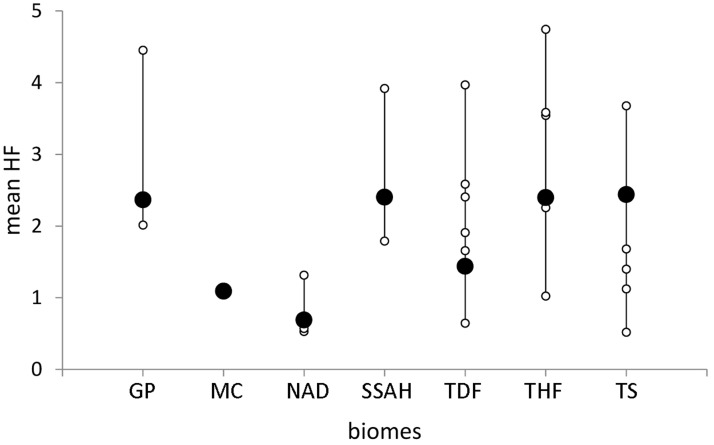
Statistical properties of the ecoregional human footprint. (a) Relationship between the mean human footprint in each of 24 Mexican ecoregions, and the standard deviation of their footprint values (*r* ^2^ = 0.99; *P* < 0.00001). (b) Relationship between mean human footprint in each ecoregion and the skewness of the distribution of footprint values (*r* ^2^ = 0.87; *P* < 0.00001). (c) Relationship between mean human footprint and the mean patch size of low footprint area in each ecoregion (*r* ^2^ = 0.84; *P* < 0.00001; in all cases the fitted curve was obtained using polynomial regression).

The statistical distribution properties of the *HF* values at the biome and ecoregional levels were similar and showed traits that are commonly observed in zero-bounded variables: On the one hand, the standard deviation of the *HF* values tended to increase with the mean footprint, until it leveled-off when mean *HF* reached a value of around 3 (Fig. 4a). Similarly, the skewness of the distribution was very high in low-footprint regions, and approached zero in the highest footprint values (Fig. 4b). That is, regions with low footprint values showed a strong *J*-shaped distribution, dominated by low-footprint areas but also harboring some small patches of high footprint sites such as cities or agricultural valleys. In contrast, as regional mean *HF* increases its distribution tends to become bell-shaped (skewness = 0), with low footprint and high-footprint areas distributed homogenously around the mean. This implies that even in regions showing the highest values of mean *HF*, the left tail of the distribution still harbors some small remaining areas with low *HF*.

The ecoregions with the largest mean patch size (more than 100 km^2^) of low-transformed vegetation cover (*HF* = 0), are located in the Sonoran and Chihuahuan deserts, Western Sierra Madre, Mediterranean California, and El Cabo. There was a negative relationship between the mean size of low footprint patches and the mean footprint (Fig. 4c). Low footprint ecoregions harbor both a higher total area (>70%) and larger continuous patches with *HF* = 0. In contrast, the mean patch size of *HF* = 0 within high-footprint ecoregions are less than 30% and 20 km^2^, respectively.

Decomposing the variance of the human footprint map into the effects of biomes and of ecoregions nested-within-biomes, we found that a significantly high amount of the variation in *HF* grid-cell values was accounted by fixed differences between biomes and between ecoregions within biomes (*P* < 0.0001 for both factors, see Table 3). The mean variance of the two levels did not differ significantly between them (*F*_6,18_ = 1.40, *P* = 0.27), indicating that variation between ecoregions within biomes is as large as between-biome variation.

**Table 3:** ANOVA.

Source	Sum Sq.	d.f.	Mean Sq.	*F*	*P*
a. Biome	474 728	6	79 121	16 484	<0.0001
b. Ecoregions within-biomes	1 013 680	18	56 316	11 732	<0.0001
c. Error	4 156 516	865 960	4.80		
Total	5 644 924	865 984			

Decomposition of the total variation in the map into (a) between-biomes variation, (b) between-ecoregions (nested within biomes) variation, and (c) the residual error, or within-ecoregions variation.

**Fig 5:**
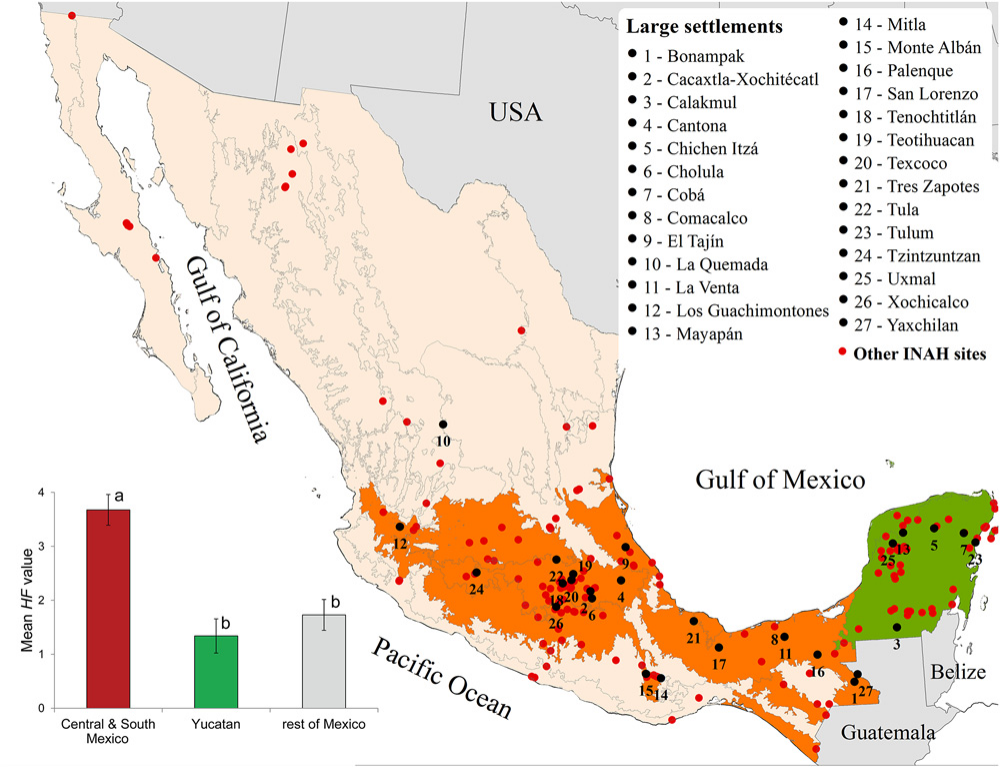
Distribution of the main pre-Hispanic settlements in Mexico. Map: Archaeological pre-Hispanic sites taken INAH (http://www.geoportal.inah.gob.mx) and major pre-Hispanic settlements taken from Kellog 2011, Sharer 1994, and Whitmore and Turner 2001 mapped over the ecoregions of Mexico (exact coordinates for each site located using GoogleEarth). The ecoregions that were most densely occupied at the time of the arrival of the first Europeans are marked in color. Inserted barchart: Mean Human Footprint value for (i) central and southern Mexico, (ii) the Yucatan peninsula, and (iii) the rest of Mexico. Different letters indicate values that differ significantly (*P* < 0.05) from each other.

Finally, we found seven ecoregions that harbored dense pre-Hispanic occupation and correspond to areas that were densely occupied before the arrival of Europeans to Mexico (Fig. 5). Two of them are in the Yucatan peninsula (Plain and Hills of the Yucatan Peninsula and Northwestern Plain of the Yucatan Peninsula) and five in central and southern Mexico (Interior Depressions, Gulf of Mexico Humid Coastal Plains and Hills, Transversal Neo-Volcanic System, Mexican High Plateau, Sierra Los Tuxtlas). To the latter list, we added the Coastal Plain and Hills of Soconusco, which, although not rich in large archaeological settlements, were an extremely important cacao agricultural area before Spanish occupation. We found a higher *HF* value in central and southern Mexico ecoregions (3.67, s.e. ±0.28) compared to the Yucatan (1.34, s.e. ±0.32) and to the rest of Mexico (1.73, s.e. ±0.29). Central and southern Mexico differed significantly from the other two regions (*t* = 5.5, *P* < 0.005; and *t* = 4.8, *P* < 0.003, respectively), but the mean *HF* in the Yucatán did not differ from that of the rest of Mexico (*t* = 0.38, *P* = 0.76; Fig. 5).

## Discussion

Despite of the long history of human use in Mexico there is still a large amount of land in the country that maintains what appears to be vegetation cover that suffered relatively low impact from human activities (*HF* = 0), mostly in Mexico’s northern deserts and along the rugged ranges of the Western Sierra Madre. The arid, harsh climate of most of the northern drylands and the steep topography and sheer inaccessibility of the sierras explain in all likelihood the existence of large proportions of these areas with minimal apparent human footprint. The large difference in *HF* values between biomes, supports the hypothesis that physical geography is a major factor controlling the anthropogenic transformation of the ecosystems. Because biomes are defined as large units with similar broad patterns of physical geography, climate, and vegetation [39], biomes that are less transformed by human action correspond, plausibly, to large regions where land transformation is either difficult or unproductive, such as abrupt sierras or deserts.

At an ecoregional level, the highest footprint values were observed along the coastal corridor of the Gulf of Mexico, from the Texas Coastal Plain in the US border all the way south to the Humid Coastal Plains in Tabasco. From this coastal corridor, an inland transect of high-footprint ecoregions runs in a general E–W direction following the ranges of the Transversal Neo-Volcanic System and the associated uplands of the Mexican High Plateau. Lastly, an area of high human footprint is found running along the Pacific coast of southern Mexico in the Coastal Plains of the Soconusco, in Chiapas. Within a biome, ecoregions share a similar large-scale physical geography, climate, and plant physiognomy; differences between them lie on aspects of local physiography, biogeographic history, and biodiversity and endemism. However, we found that the ANOVA variation of ecoregions within-biomes is as important as that among biomes, a fact that supports our second hypothesis, namely that, at this scale, historical geography has played an important role in the transformation of the original land surface. For example, within the Tropical Humid Forests biome, the Plains and Hills of the Yucatan Peninsula have a mean *HF* value of 1.02, while the Sierra de Los Tuxtlas, one of the most intensely deforested regions of Mexico, has a mean *HF* value of 4.74, the highest for any ecoregion in the country (Table 2). Both areas have similar climate and vegetation, the main difference between them lies in their past histories of natural resource use and land clearing: While the Yucatan forests were depopulated after the collapse of the Classic Maya and have maintained relatively low population densities since, the Los Tuxtlas region suffered intense deforestation in the last century as a result of a growing demand for tropical agricultural land and pastures [40]. Similarly, within the Tropical Dry Forests biome the Sierras and Plains of El Cabo show one of the lowest footprint values of Mexico (*HF* = 0.64) while the Gulf of Mexico Dry Coastal Plains and Hills (often known as the Tamaulipan dry forests) have one of the largest (*HF* = 3.97). Similar vast differences between ecoregions that are climatically and geographically alike can be seen for most other biomes in Mexico.

The accelerated development of Mexico during the 20^th^ Century partially explains this pattern: The coastal plains of the Gulf of Mexico harbor two large industrial areas (Monterrey in the north and Coatzacalcos in the south), Mexico’s largest ports, its oldest oil industry, and some of its most productive agricultural land. The inland transect of fertile basins and valleys that runs parallel to the transversal volcanic ranges harbors an urban chain of Mexico’s largest cities: Puebla, Mexico City, Toluca, Querétaro, León, Guanajuato, and Guadalajara, among others. The agricultural coastal plains of Sonora, Sinaloa, and Nayarit—cradle of Mexico’s “green revolution”—contain some of its most productive grain and produce fields. But an interpretation of these high footprint areas and of the human footprint pattern in Mexico based solely on a contemporary vision might be missing some potential causes of the spatial pattern of land use in the country.

**Fig 6:**
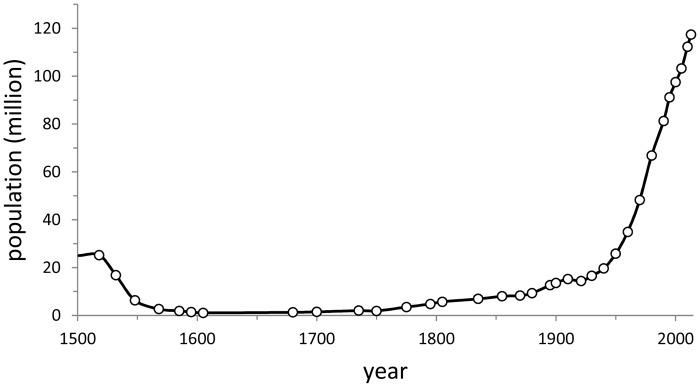
Population in Mexico between 1521 and present. Data compiled from Borah and Cook 1969, McCaa 2000, Benítez Zenteno 1961, and official population censuses available at www.inegi.gob.mx.

Borah and Cook [22], and references therein, estimated the total pre-conquest population of what is now the Mexican nation in around 25 million. Although many researchers may sustain different estimates (e.g., [41, 42]), what is undoubtedly true is that when the Spaniards reached Mexico they found a densely inhabited territory and a highly transformed landscape [23] where somewhere between 10 and 25 million people lived (Fig. 6). Large, dense civilizations had risen and collapsed for millennia all over central Mexico, and had left behind their footprint on the environment in terms not only of large settlement centers, but also in terms of cleared land, terraced landscapes, irrigation systems, and transformed forests [38,43]. In the century that followed the Spanish conquest, the population of Mexico plummeted to around a million as the indigenous people succumbed to new diseases, wars with the Europeans, the collapse of local economies, and the *encomienda* system, in one of the most catastrophic population collapses known in history (Fig. 6) [44]. The demographic tragedy seriously affected agriculture and land use in Mexico; the cropping systems of the indigenous peoples could not be sustained with such losses in labor, leading to the abandonment of many cultivated landscapes [22] and opening the way for land appropriation by the Spanish of the depopulated land and the development of the *hacienda* system of large estates managed by the new ruling class [45].

Much of the land that was once under indigenous cultivation was put to a new use (e.g., [26]). Although the crops and the agricultural techniques changed under Spanish rule, the occupation of the conquered territory followed the patterns of pre-Hispanic land use. Cattle and sheep production was introduced to the Gulf Coast by burning forest, wetlands, and former tropical orchards, altering indigenous agricultural landscapes to encourage the growth of grasses. Plantation crops emerged in the Gulf of Mexico, including sugarcane, first, followed later by cacao, cotton, and tobacco, all New World crops that found a fast growing demand in Europe during the Spanish Colony. Similar tropical-plantation development took place in the Soconusco region in Chiapas near the Guatemalan border, where the fertile coastal lowlands had been an important cacao-growing area in pre-Hispanic times (e.g., [46]), but underwent an explosive growth into the mountain slopes in the 19^th^ and 20^th^ Centuries, driven mostly by growing markets for cacao and coffee [47].

In central Mexico and the Gulf Coast there is a remarkable coincidence of highly impacted areas with the historic distribution of pre-Hispanic settlements and civilizations. As a general rule, the areas most transformed by human activities lie in the Gulf Coast and follow the E–W chain of cities along the central highlands, showing a marked spatial coincidence with the number of large pre-Hispanic settlements that existed at the time of Spanish arrival in Mexico in the early 16^th^ Century.

In contrast, no significant spatial association was found in the Lowland Maya region of Yucatan between the footprint values of each ecoregion and the number of pre-Hispanic settlements in them. This apparent low footprint of past Maya civilizations has been analyzed in detail by Arturo Gómez-Pompa and his collaborators [48], who demonstrated that many forests of the Maya region are really a mosaic of remnants of ancient orchard-gardens, and that most of the Maya “wilderness” is really formed by new growth over old agricultural plots and tropical orchards, recolonized by forests after the collapse of the classic Maya a few centuries before the Spanish conquest.

Finally, in the country’s arid north a number of intensive processes of land-use change took place almost entirely during the 20^th^ century to promote the development of new irrigation districts on the fertile alluvial plains of dryland rivers. Undertaken in the 1930s, shortly after the end of the Mexican Revolution, in large part to create new jobs and open new agricultural frontiers, the footprint of these massive projects is clearly visible in the low basin of the Nazas River in the heart of the Chihuahuan Desert; along the coast of Sonora and Sinaloa on the lowland plains of the Baluarte, Fuerte, Mayo, and Yaqui rivers; in the Mexicali Valley on the Colorado River deltaic plains; and on the Santo Domingo basin in Baja California Sur. It was basically modern technology—in the form of deep-well drilling, river dams, electrification, refrigeration and air conditioning—what allowed the colonization of these areas. Together with some industrial cities in Mexico’s northern ecoregions—such as Monterrey, Monclova, Torreón, Juárez, or Tijuana—these dryland areas of high human footprint are mostly the result of accelerated expansion during the 20^th^ Century as the country’s development efforts started shifting towards its northern drylands.

Although, according to existing land-use maps, much of the low footprint dryland regions are classified as harboring native vegetation cover, these ecosystems have been used for centuries and many of them have suffered intense biological changes as a result [49,50]. Hidden anthropogenic change has been reported for the Chihuahuan and Sonoran deserts, where the introduction of cattle produced the invasive growth of native *Opuntia* cacti, changing the dominance structure of the native plant communities [51] but still allowing them to be seen by some as pristine or “wild”. The low *HF* values found for these ecoregions using map layers contrast with official statistics on rangeland degradation [50], where more than 50% of the area with native vegetation cover in the northern drylands is officially reported as overgrazed or impacted by cattle-induced shrub encroachment, a fact that highlights the large impact that the introduction of grazing animals from Europe brought to the region and how this impact is often hidden behind the apparent permanence of native vegetation. Thus, the method we used and the resulting map might be downplaying the true magnitude of anthropogenic transformations in the lower footprint categories. These hidden impacts on native ecosystems and biodiversity need to be considered in more detail in future research to be able to give a more accurate picture of the impact of anthropogenic influence on a region’s biological diversity and ecosystem services.

Finally, the Human Footprint algorithm was able to quantify with precision the size and location of continuous patches of (relatively) well-preserved ecosystems. Most of the large patches of low-transformed vegetation cover lie in Mexico’s arid north, where, not surprisingly, most of the new large protected areas decreed by the Federal Government during the last decades area found. The more anthropogenically-impacted ecoregions of central and southern Mexico harbor in general smaller patches of well-preserved vegetation cover, but, despite this, a number of these extant patches still can be found. The approach we are using in this analysis can inform and guide future efforts for conservation planning at a national level.

The human footprint model quantifies in detail patterns that are often known, or suspected, but not amenable to rigorous statistical analysis and hypothesis testing. The model was able to identify extant patches of relatively well-preserved vegetation that may inform future initiatives of biodiversity conservation. The model is also able to detect large expanses of land that have been severely transformed by human action but that harbor relatively low population densities, such as the irrigated arid farmlands in the coast of the Sonoran Desert, and may provide a tool to understand large-scale transformations of the environment that is substantially more powerful than, say, population density, distribution of urban areas, or analysis of land-use changes alone.

The spatial spread of the human footprint in Mexico is both the result of the limitations imposed by physical geography to human development at the biome level, and, within different biomes, of the environmental legacy of past civilizations and population collapses, including, but not exclusively restricted to, the 20^th^ Century demographic explosion. Throughout Mexico, and very especially in the central part of the country, the current spatial distribution of highways, farms, and cities still reflects civilizations, technologies, and societal interactions of the past.
