# Correction: The Human Footprint in Mexico: Physical Geography and Historical Legacies

**DOI:** 10.1371/journal.pone.0128055

**Published:** 2015-05-04

**Authors:** Charlotte González-Abraham, Exequiel Ezcurra, Pedro P. Garcillán, Alfredo Ortega-Rubio, Melanie Kolb, Juan E. Bezaury Creel

The legends for Figs 3 and 4 have been incorrectly switched. Please view Figs 3 and 4 with their correct legends here.

**Fig 3:**
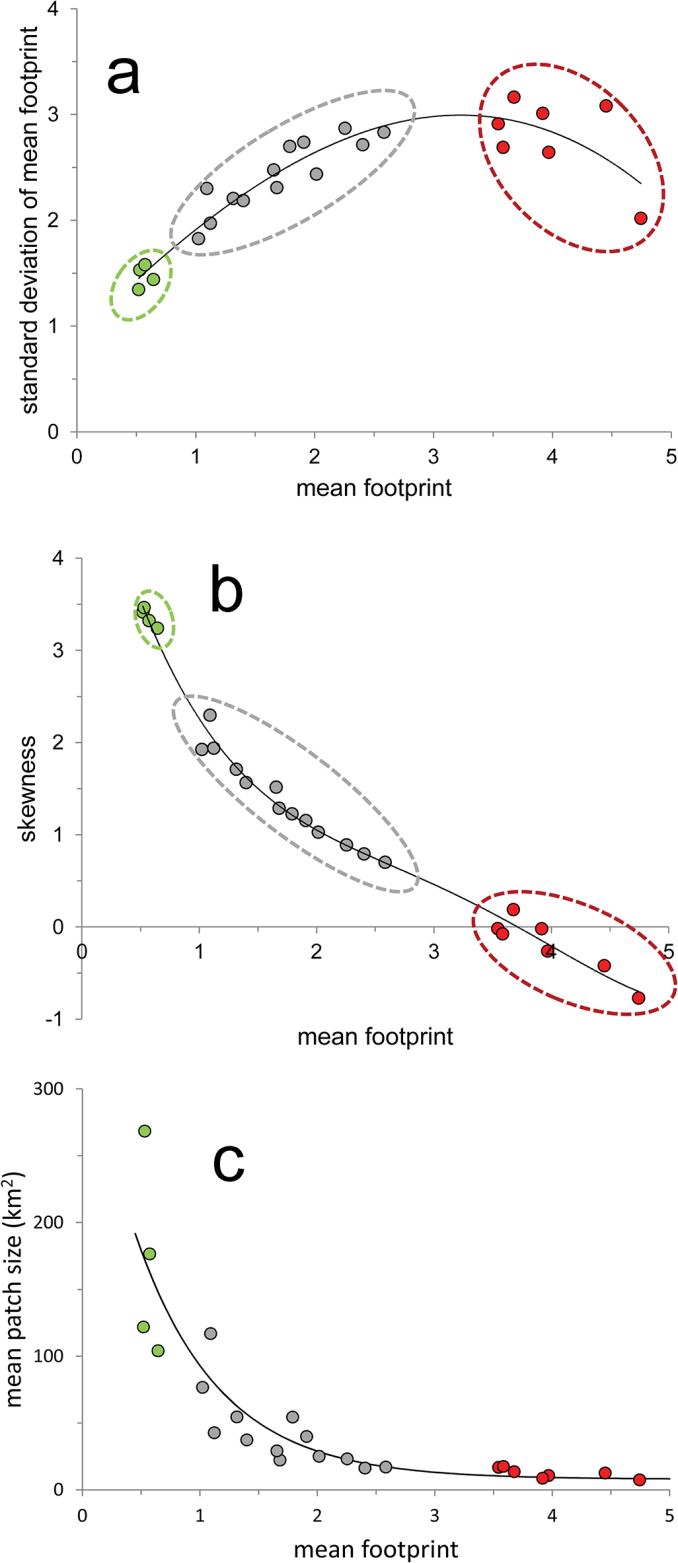
Statistical properties of the ecoregional human footprint. (a) Relationship between the mean human footprint in each of 24 Mexican ecoregions, and the standard deviation of their footprint values (*r* ^**2**^ = 0.99;*P* < 0.00001). (b) Relationship between mean human footprint in each ecoregion and the skewness of the distribution of footprint values (*r* ^**2**^ = 0.87; *P* < 0.00001). (c) Relationship between mean human footprint and the mean patch size of low footprint area in each ecoregion (*r* ^**2**^ = 0.84; *P* < 0.00001; in all cases the fitted curve was obtained using polynomial regression).

**Fig 4:**
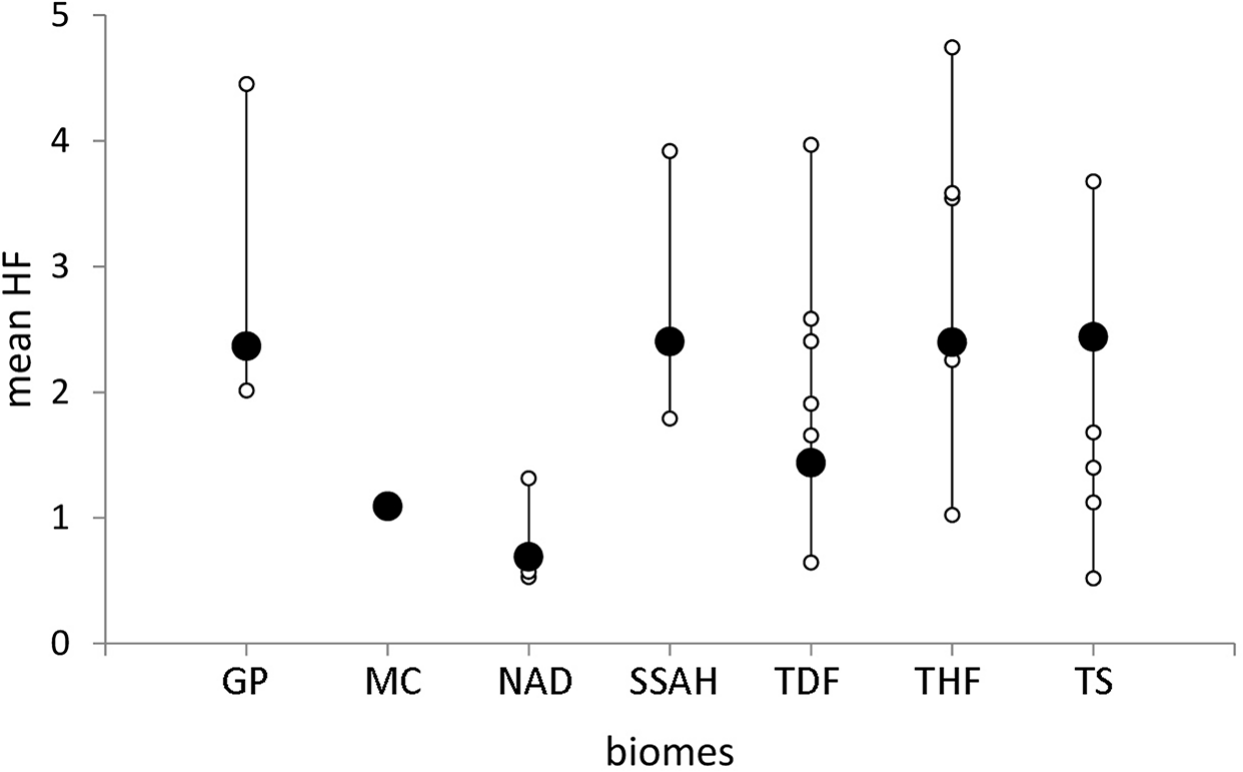
Ecoregional footprint nested within biomes. Large differences in ecoregional *HF* values (white points) were observed within most biomes (black points). Biomes names as follow Great Plains (GP), Mediterranean California (MC), North American Deserts (NAD), Southern Semi-Arid Highlands (SSAH), Temperate Sierras (TS), Tropical Dry Forests (TDF) and Tropical Humid Forests (THF).
